# Does health literacy influence health-related lifestyle behaviors among specialists of health management? A cross-sectional study

**DOI:** 10.1186/s12875-024-02263-1

**Published:** 2024-01-20

**Authors:** Shunsuke Kinoshita, Nobutaka Hirooka, Takeru Kusano, Kohei Saito, Ryutaro Aoyagi

**Affiliations:** 1 Department of General Internal Medicine, Saitama Medical University, Iruma-gun, Japan; 2 Department of Community Medicine, Saitama Medical University, Iruma-gun, Japan

**Keywords:** Health literacy, Health-related lifestyle behavior, National health promotion

## Abstract

**Background:**

Noncommunicable diseases (NCDs), such as health-related lifestyle diseases, are the leading cause of mortality and societal and economic burdens. Poor lifestyle behaviors, which are modifiable to improve health, can cause diseases, including NCDs. Health literacy has been recognized as an important determinant of health, and studies have shown that higher health literacy is associated with better health outcomes and positive health-related behaviors. However, few studies have investigated the association between health literacy and health-related lifestyle behaviors to understand the mechanistic link between them. Thus, this study investigated the extent to which health literacy at different levels influences health-related lifestyle behaviors.

**Methods:**

A cross-sectional study was conducted among Japanese health management specialists (*N* = 1,920). Functional, critical, and communicative health literacy were measured. Lifestyle behaviors (exercise, diet and nutrition, sleep, rest, smoking, and alcohol intake), in line with the Japanese National Health Promotion Program, were assessed and calculated into a total cumulative score of health-related lifestyle behaviors. Moreover, we analyzed the associations between the three levels of health literacy and lifestyle behaviors using regression analyses by adjusting for socio-psycho-demographic factors.

**Results:**

Multiple linear regression analyses showed a significant association between the Japanese version of the European Health Literacy Survey Questionnaire and total health-related lifestyle scores (standardized β = 0.160, *p* < 0.001, *R*^2^ = 0.136) after adjusting for sociodemographic factors. Similarly, the association between communicative and critical health literacy and the total health-related lifestyle scores was significant (standardized β = 0.122, *p* < 0.001, *R*^2^ = 0.125). The analysis indicated that individuals who had higher level of health literacy (critical and communicative) than functional health literacy (Japanese version of the Newest Vital Sign score) had higher health-related lifestyle behaviors.

**Conclusions:**

A higher level of health literacy is associated with health-related lifestyle behaviors. Health literacy can be a target for interventions to achieve the national goal of lifestyle-related disease prevention and control.

## Background

Many studies have shown that poor lifestyle behaviors cause diseases, such as cardiovascular diseases [1, 2], cancer [3, 4], chronic respiratory diseases [5–7], and diabetes [8–10]. Noncommunicable diseases (NCDs), such as health-related lifestyle diseases, are the leading cause of mortality and societal and economic burdens [11–13]. In addition, many studies have shown that appropriate interventions for health-related lifestyles reduce the incidence of NCDs [14–16]. Thus, health-related lifestyle behaviors are critical targets for interventions to prevent such diseases and reduce the cost of care for them. Besides the medical and societal benefits of improving health-related lifestyle behaviors, studies support that healthy lifestyle behavior benefits people psychologically by showing reduced stress, happiness, higher quality of life, and meaning of life [17–19]. Similar to NCDs, these psychological aspects are considered of national interest in public health policies of multiple countries [20, 21]. Because of the multiple benefits of better health-related lifestyle behaviors, such behaviors are the main target for achieving national health promotion goals in many countries [22–24]. The World Health Organization also supports governmental actions to reduce the burden of NCDs through lifestyle intervention [25].

Health literacy is defined as cognitive and social skills that determine a person’s motivation and ability to gain access to, understand, and use information in ways that promote and maintain good health [26]. Health literacy has been recognized as an important determinant of health, and studies have shown that higher health literacy is associated with better health outcomes and positive health-related behaviors [27–29]. In relation to NCDs, health literacy is the key concept for the prevention, clinical treatment, and control of diseases for public health purposes. Studies have shown that better health outcomes related to NCDs were brought about by health literacy interventions [30, 31].

Despite studies reporting the effects of health literacy on healthier outcomes, the association and causal relationship between health literacy and health-related lifestyle behaviors have been investigated in a limited area and context. Nutbeam categorized health literacy into three levels—functional, communicative, and critical—but many studies have focused on only one level of health literacy [26]. In addition to the narrow aspect of health literacy measurements in the studies, the validity of such measurements was generally insufficient [32]. Moreover, the target populations in previous studies were limited; that is, although the study target population is expanding, majority of the studies were performed in the US and European countries. Additionally, a high proportion of research on health literacy has focused on clinical settings [33, 34]. Thus, there is little evidence to show the association between health literacy and health behavior among the most rapidly aging societies, including Japan, in nonclinical settings.

Healthcare personnel play an important role in health promotion, as they often provide advice on healthy lifestyles. Studies have shown that healthcare personnel are more likely to encourage healthy lifestyle behaviors and provide preventive counseling in clinical settings when they engage in such behaviors [35, 36]. However, although it is crucial for healthcare personnel to maintain healthy lifestyle behaviors to look after their own health, some studies have suggested that health professionals may not practice the expected health-related lifestyle behaviors [37, 38]. Studies have also suggested that more health literacy training is needed for healthcare professionals.

At present, there is little research investigating the association between health literacy and health-related lifestyle behaviors in the context of national health promotion, especially multiple lifestyle behaviors rather than a single specific behavior among healthcare personnel. Investigating the effect of different levels of health literacy on health-related lifestyle enables us to better understand the relation between health literacy and health outcomes and may suggest an effective intervention approach in health promotion practice. This study thus investigated the extent to which health literacy at different levels influences health-related lifestyle behaviors among Japanese specialists of health management, in line with national health promotion to test the hypothesis that higher level of health literacy is required to show the association with health-related lifestyle behaviors.

## Methods

### Study design

This was a cross-sectional survey of professional health management specialists. The study participants completed a survey on demographic data, health literacy, and health-related lifestyle behaviors. All questionnaires were sent by mail and administered at the time when the study participants were enrolled after reading the explanation of the study and returning the signed documents of the informed consent to participate in the study. This study was approved by the Ethics Committee of Saitama Medical University (ID 926, 2020).

### Study participants

Study participants were health management specialists certified by the Japanese Association of Preventive Medicine for Adult Disease (JAPMAD) [39]. This certificate program for health management specialists was sponsored by the Ministry of Education, Culture, Sports, Science and Technology, Japan. These specialists are expected to engage in the community in which they live to conduct health promotion workshops. Specialists in health management are certified through multiple courses of study. Candidates studied various aspects of the course, including health promotion, lifestyle-related diseases, mental health, nutrition, environment and health, physical activity and exercise, emergency medicine, life support, and the healthcare system. It takes on average 4 months to complete the course. The candidates passed the final written examination to be able to register as specialists. All of the specialists who met the criteria were contacted by mail on August 1, 2020, to participate in the study. The data was collected from August 1st, 2020 to March 31st, 2021. The inclusion criteria of the study included continued participation in lifelong education provided through JAPMAD, and provision of written informed consent. There were no exclusion criteria. Among the individuals who met the inclusion criteria (*n* = 4,530), 1,920 (response rate: 42.4%) agreed to participate in the study, all of whom were included in the analysis.

### Variables and measurements

Variables collected in this study included demographic data, health literacy, and health-related lifestyle behaviors. Health literacy was measured using the Communicative and Critical Health Literacy (CCHL) scale [40], the European Health Literacy Survey Questionnaire (EU-HLS-Q47) [41], and the Newest Vital Sign (NVS-J) [42]. Both Japanese version of EU-HLS-Q47 (EU-HLS-Q47-J) [43] and NVS (NVS-J) [44] were validated previously among Japanese.

CCHL comprises three items on communicative health literacy and two items on critical health literacy. Communicative health literacy asked if the study participants were able to (1) collect health-related information from various sources, (2) extract the information they wanted, (3) understand and communicate the obtained information, (4) consider the credibility of the information, and (5) make decisions based on the information specifically in the context of health-related issues. Each item was rated on a five-point Likert scale ranging from 1, “strongly disagree,” to 5, “strongly agree.” The NVS-J assesses functional health literacy and consists primarily of questions requiring study participants to read and interpret numerical facts by reading a standard nutritional label. The EU-HLS-Q47-J asked about the functional, communicative, and critical level of health literacy in Japanese ranging from 1, “very difficult” to 4, “very easy,” in addition to 5, “don’t know.”

Lifestyle behaviors regarding eating/dieting, exercise/physical activity, sleep, rest, smoking, and alcohol intake were assessed in the questionnaire as recommended by the Japan’s National Health Promotion Program in the 21st Century (HJ21) [45]. There were 11 health-related lifestyle questions, of which four were two-scaled (“intention to maintain ideal weight,” “exercise,” “manage lifestyle to prevent disease,” and “smoking”). For these items, a score of “1” was assigned for an unhealthy lifestyle behavior and “4” for healthy lifestyle behavior. This mechanism was used to ensure impartiality. Questions regarding alcohol intake included type of alcohol, amount of alcohol consumed, and frequency of drinking. This information indicated if participants drank more than the amount recommended (less than 20 g per day on average) by the [45]. Then, a score of “1” was assigned if participants drank more than a recommended level and “4” if they drank less than 20 g per day on average. The remaining six health-related habits (“reading nutritional information labels,” “maintaining a balanced diet in daily life,” “intention to exercise,” “stress,” “rest,” and “sleep”) were assessed using four scales. Thus, “4” (most favorable) to “1” (least favorable) were assigned to these variables. We then added the values of each answer to the questions on the participants’ health-related lifestyle behaviors as clustered health-related lifestyle scores. Thus, the lowest and highest possible scores were 11 and 44.

### Analysis

Descriptive statistics (mean, average, standard deviation, and range) were used to describe characteristics of the study participants. Pearson’s correlational analyses were performed among the three measurements of health literacy (NVS-J, CCHL, and EU-HLS-Q47-J). Multiple linear regression tests were performed to explain the health-related lifestyle scores based on health literacy. Three different models were fit to adjust for covariates in all three health literacy measurements based on the theoretical model by Sun et al. [46]. The first model (Model 1) was a simple regression between health literacy and lifestyle behaviors. Model 2 included age, sex, income, education, marital status, and family as covariates in the regression analysis. Model 3 included disease status (presence or absence of diabetes mellitus, hypertension, dyslipidemia, cancer, and obesity) in the regression analysis. Statistical significance was set at *p* < 0.05. The list-wise deletion method for missing data was used while missing data was less than 0.5%. All statistical tests were two tailed. IBM SPSS Statistics (version 26.0. Armonk, NY, USA) was used for all analyses.

## Results

Table 1 presents the demographic characteristics of the study participants. Overall, 1,920 certified health management specialists were included in this study. More women (*N* = 1,181; 61.5%) participated in the study than men. The age range of study participants was from 22 years to 93 years. More than two-thirds of them were married, and three-quarters lived with family. Majority of them (71.9%) received education higher than high school. The averages (standard deviation) of NVS-J, CCHL, and EU-HLSQ47-J were 5.02 (1.67), 18.55 (3.65), and 30.43 (8.36), respectively.

**Table 1:** Demographic characteristics of the study participants

Characteristics	Total (*N* = 1,920)
Sex; N (%)	
Male	739 (38.5)
Female	1,181 (61.5)
Age range; N (%)	
< 30 years	24 (1.3)
30–39 years	109 (5.7)
40–49 years	312 (16.3)
50–59 years	596 (31.0)
60–69 years	552 (28.8)
70–79 years	277 (14.4)
≥ 80 years	50 (2.6)
Age; Average years (standard deviation [SD])	58.5 (21.5)
Education; N (%)	
Junior high school	27 (1.4)
High school	511 (26.6)
Professional training college	331 (17.2)
College	296 (15.4)
University/Graduate school	755 (39.3)
Marital status; N (%)	
Married	1,419 (73.9)
Family; N (%)	
Yes	1,585 (82.6)
Income (million yen/year); N (%)	
< 200	180 (9.4)
200–600	1,017 (53.0)
> 600	717 (37.3)
Intention to keep ideal weight; N (%)	
Yes	1,583 (82.4)
No	337 (17.6)
Managing lifestyle for disease prevention; N (%)	
Yes	1,700 (88.5)
No	220 (11.5)
Reading nutritional information labels; N (%)	
Always	691 (36.0)
Often	874 (45.5)
Rarely	277 (14.4)
Very rarely	78 (4.1)
Maintaining a balanced diet in daily life; N (%)	
Always	1,087 (56.6)
Often	677 (35.3)
Rarely	142 (7.4)
Very rarely	14 (0.7)
Intention for exercise; N (%)	
Always	897 (46.7)
Sometimes	756 (39.4)
In the past	214 (11.1)
Never	52 (2.7)
Adequate exercise; N (%)	
Yes	1,282 (66.8)
No	638 (33.2)
Excessive alcohol intake; N (%)	128 (6.7)
Smoking (%)	
Current	381 (19.8)
Past	95 (4.9)
None	1,435 (74.7)
Stress; N (%)	
High	353 (18.4)
Moderate	1,030 (53.6)
Low	451 (23.5)
None	84 (4.4)
Rest; N (%)	
Satisfactory	451 (23.5)
Adequate	1,027 (53.5)
Not adequate	388 (20.2)
Not satisfactory	51 (2.7)
Sleep; N (%)	
Satisfactory	434 (22.6)
Adequate	1,100 (57.3)
Not adequate	370 (19.3)
Not satisfactory	14 (0.7)
Total health-related lifestyle score; average (SD) 95% confidence interval	35.8 (4.15) [35.7–36.0]

Table 2 shows results of the correlation analyses of the three health literacy measurements. These analyses found a statistically significant correlation between the EU-HLS-Q47-J and the CCHL. While the correlation between the NVS-J and CCHL was significant, the strength of the correlation was low (*r* = 0.070). There was no significant correlation between NVS-J and EU-HLS-Q47-J.

**Table 2:** Correlational coefficient among the NVS-J, CCHL, and EU-HLS-Q47-J

	NVS-J	CCHL	EU-HLS-Q47-J
NVS-J	1.0	-	-
CCHL	0.070*	1.0	-
EU-HLS-Q47-J	0.027	0.318*	1.0

Notes: NVS-J: Japanese version of the New Vital Sign; CCHL: Communicative and Critical Health Literacy scale; EU-HLS-Q47-J: Japanese version of the European Health Literacy Survey Questionnaire; * *p* < 0.01

The simple regression (Model 1) or multivariable regression (Model 2 and 3) analyses after adjusting for the predetermined variables (age, sex, income, education, status of marriage, family, and lifestyle-related diseases) did not show any significance for NVS-J in explaining total lifestyle behaviors (*p* = 0.80, 0.10, and 0.15 for Models 1, 2, and 3, respectively). Tables 3 and 4 show the results of the regression analyses between the two different types of health literacy (EU-HLS-Q47-J and CCHL) and lifestyle behaviors. In both EU-HLS-Q47-J and CCHL analyses on lifestyle behaviors, health literacy significantly explained the variability of total lifestyle behaviors (all *p* < 0.001). Multicollinearity was assessed in each of the model using VIF, which was less than 2 and no sign of multicollinearity was found.

**Table 3:** Results from the multiple regression analyses of EU-HLS-Q47-J and total health-related lifestyle score

	Unstandardized b (SE)	Standardized β	*p*	R^2^
Model 1	0.094 (0.011)	0.189	< 0.001	0.035
Model 2	0.078 (0.011)	0.158	< 0.001	0.133
Model 3 Age Sex Education Marital status Family Income Disease status	0.079 (0.011) 0.112 (0.008) 0.489 (0.189) 0.186 (0.072) − 0.508 (0.245) 0.040 (0.274) − 0.347 (0.154) − 0.398 (0.137)	0.160 0.319 0.057 0.057 − 0.055 0.004 − 0.053 − 0.065	< 0.001	0.136

Notes: EU-HLS-Q47-J: Japanese version of the European Health Literacy Survey Questionnaire; SE: standard error; b: unstandardized coefficients; β: standardized coefficients, *R*^2^: coefficient of determinant. Model 1 was adjusted for no variable; Model 2 was adjusted for age, sex, income, education, marital status, and family; Model 3 was adjusted for lifestyle-related disease (presence or absence of diabetes mellitus, hypertension, dyslipidemia, cancer, and obesity) in addition to the variables adjusted in Model 2. * indicates *p* < 0.05

**Table 4:** Results from the multiple regression analyses of CCHL and total health-related lifestyle score

	Unstandardized b (SE)	Standardized β	*p*	R^2^
Model 1	0.164 (0.026)	0.145	< 0.001	0.020
Model 2	0.142 (0.024)	0.125	< 0.001	0.124
Model 3 Age Sex Education Marital status Family Income Disease status	0.138 (0.025) 0.114 (0.008) 0.527 (0.189) 0.219 (0.072) − 0.528 (0.247) − 0.040 (0.275) − 0.349 (0.155) − 0.338 (0.189)	0.122 0.323* 0.062* 0.068* − 0.057* − 0.004 − 0.053* − 0.040	< 0.001	0.125

Notes: CCHL: Communicative and Critical Health Literacy scale; SE: standard error; b: unstandardized coefficients; β: standardized coefficients, *R*^2^: coefficient of determinant. Model 1 was adjusted for no variable; Model 2 was adjusted for age, sex, income, education, marital status, and family; Model 3 was adjusted for lifestyle-related disease (presence or absence of diabetes mellitus, hypertension, dyslipidemia, cancer, and obesity) in addition to the variables adjusted in Model 2. * indicates *p* < 0.05

## Discussion

This study indicated that a higher level, but not a basic level, of health literacy was associated with healthy lifestyle behaviors. Reports have demonstrated an association between health literacy and health-related behaviors. Limited health literacy has been shown to be associated with unhealthy lifestyle behaviors [47–50]. Other studies have demonstrated that higher health literacy is related to increased exercise and fruit and vegetable consumption [29, 51, 52]. However, not all studies on health literacy have found such a relationship. Additionally, studies tended to investigate disease populations, such as patients with diabetes, rather than the general population. Showing the association between higher level, not basic level of health literacy, and wide spectrum lifestyle behaviors among people who do not have specific diseases in this study expands the scientific knowledge. In particular, individuals who had gained knowledge and skills of health promotion in line with HJ21 as the target population were directed toward future actions for national health promotion.

While both the EU-HLS-Q47-J and CCHL explained health-related lifestyle behaviors statistically significantly, the NVS-J was not associated with health-related lifestyle behaviors in this study. Various instruments have been developed to improve health literacy. The NVS-J primarily measures the functional level of health literacy, assessing basic reading and writing skills to function effectively in everyday situations. The EU-HLS-Q47-J and CCHL assess more complex health literacy, including communicative and critical health literacy, for practical application in everyday life. Progression from basic and functional health literacy to communicative and critical health literacy requires multiple factors such as advanced cognitive skills, literacy, social and communication capacity, and self-efficacy [26]. Thus, the functional level of health literacy may not be adequate to change behaviors in terms of health. This is supported by the results that only communicative and critical health literacy, but not functional literacy, were associated with health-related lifestyle behaviors in the study.

There are several hypothesized mechanistic links between health literacy and health actions and outcomes in prior studies [53–55]. However, most of these target limited health literacy and patients with certain diseases. None of them provide a clear causal link between health literacy and healthy lifestyle behaviors among populations that are not restricted to narrow disease entities. Baker proposed that health literacy is causally linked to improved health outcomes through several factors, such as new knowledge, positive attitudes, self-efficacy, and behavior change [54]. Paasche-Orlow and Wolf illustrated the causal pathway between limited health literacy and health outcomes, where patient and extrinsic factors—including motivation, self-efficacy, problem-solving skills, resources, and support technology—mediate this relationship [53]. Since the study population is largely well-educated and specialized in health management, this character may affect the mediating and/or moderating effect of health literacy on health-related lifestyle behaviors through these factors among the health professional. Studies on health literacy interventions reported improved health outcomes. While a causal pathway between health literacy and health-related lifestyle behaviors is needed to determine how and what factors affect the relationship, our finding—that a higher level of health literacy is related to health-related lifestyle behaviors—implies that future interventions should target improvements in levels of health literacy. Whether a functional level of health literacy underpins a higher level of health literacy, and/or is necessary for acquiring higher levels when placing interventions to achieve better health outcomes, is of scientific interest considering effective health literacy interventions for a larger population.

Health literacy seems to be the key target for national health promotion, based on existing evidence and our results. The World Health Organization recognized the efforts to raise health literacy as crucial in the 2030 agenda for sustainable development [56]. Many countries, including Japan, emphasize the importance of health literacy in their action plans for national health promotion in an aging environment [57, 58]. There is ample evidence that a healthy lifestyle prevents many diseases such as obesity, diabetes, cardiovascular disease, stroke, or cancers. In addition to the causal relationship between healthy lifestyle and disease prevention, our finding—that higher health literacy helps people improve health-related lifestyle behaviors—supports the national health promotion program that incorporates health literacy intervention into the core target. In the next term of HJ21 beginning from year 2024 [59], lifestyle modification remain the main goals to avoid lifestyle-related diseases, health literacy of communicative and critical level can be the target skills to achieve the goal of HJ21.

This study has several strengths. First, it measured the different types and categories of health literacy. Many studies have emphasized the importance of the method of health literacy measurement due to lack of robustness in previous health literacy studies [32, 60]. All categories of health literacy, including functional, communicative, and critical health literacy in the study, as well as the different effects on lifestyle, are scientifically important evidence. Second, the measurements are in line with the national health promotion policy, which potentially allows us to apply the effect of health literacy to a large population.

This study has a few limitations as well. First, the sampling method did not involve random sampling of the population. While the study’s participation rate was relatively high (42.4%) and age and sex were comparable between the population and study participants (not reported), does not allow application of the results to the general population. Generalizability to other populations or the general population requires a more robust sampling methodology, which is our future target. Second, the measurements were self-administered and may have included report bias.

## Conclusion

Health literacy of a higher (communicative and critical) level, but not the functional level, is associated with health-related lifestyle behaviors of health professionals. Therefore, public health practices should target a higher level of health literacy as an intervention to achieve the national goal of lifestyle-related disease prevention and control.

## Data Availability

The datasets used in the current study are available from the corresponding author upon reasonable request.

## Abbreviations

CCHL: Communicative and critical health literacy
EU-HLS-Q47-J: Japanese version of the European Health Literacy Survey Questionnaire
HJ21: Japan’s National Health Promotion Program in the 21st Century
NCDs: Noncommunicable diseases
NVS-J: Japanese version of the Newest Vital Sign
